# Effects of care of multimorbid patients in general practices by advanced practice nurses (FAMOUS): study protocol for a nonrandomized controlled trial

**DOI:** 10.1186/s12913-023-09460-1

**Published:** 2023-05-17

**Authors:** Renate Stemmer, Britta Büchler, Matthias Büttner, Christina Dera-Ströhm, Joachim Klein, Emilio Gianicolo

**Affiliations:** 1 Faculty of Health Care and Nursing, Catholic University of Applied Sciences Mainz, Mainz, Germany; 2 Institute of Medical Biostatistics, Epidemiology and Informatics (IMBEI), University Medical Center of the Johannes Gutenberg University Mainz, Mainz, Germany; 3 Institute of Clinical Physiology, National Research Council, Lecce, Italy

**Keywords:** Primary health care, Multimorbidity, Advanced practice nurses, Evidence-based nursing, Person-centred care, Nonrandomized controlled trial

## Abstract

**Background:**

Multimorbidity is a common phenomenon among patients treated in general practices. Key challenges within this group include functional difficulties, polypharmacy, treatment burden, fragmentation of care, reduced quality of life and increased health care utilization. These problems cannot be solved in the short consultation time of a general practitioner (GP) since there is an increasing shortage of GPs. In many countries, advanced practice nurses (APNs) are successfully integrated into primary health care for multimorbid patients.

The objective of this study is to examine whether the integration of APNs in the primary care of multimorbid patients in Germany leads to optimized care of the target group and to a reduction in the workload of the GPs.

**Methods:**

The intervention includes the integration of APNs into the care for multimorbid patients in general practice for twelve months. Qualifications for APNs include a master's level academic degree and 500 hours of project-specific training. Their tasks include in-depth assessment, preparation, implementation, monitoring and evaluation of a person-centred and evidence-based care plan.

In this nonrandomized controlled trial, a prospective multicentre mixed methods study will be performed. The main inclusion criterion was the cooccurrence of three chronic diseases. For data collection in the intervention group (*n* = 817), routine data from health insurance companies and association of statutory health insurance physicians (ASHIP) will be used, as well as qualitative interviews. In addition, the intervention will be assessed through documentation of the care process and standardized questionnaires using a longitudinal design. The control group (*n* = 1634) will receive standard care. For the evaluation, routine data from health insurance companies are matched at a ratio of 1:2.

Outcomes will be measured using emergency contacts and GP visits, treatment costs, health status of the patients and the satisfaction of parties involved.

The statistical analyses will include Poisson regression to compare outcomes between the intervention and control groups. Descriptive and analytical statistical methods will be used in the longitudinal analysis of the intervention group data. Cost analysis will compare total costs and subgroup costs between the intervention and control groups. Qualitative data will be analysed using content analysis.

**Discussion:**

Challenges to this protocol could include the political and strategic environment as well as the planned number of participants.

**Trial registration:**

DRKS00026172 on DRKS.

## Administrative information

The structure of this report corresponds to the SPIRIT checklist. Thus, the administrative data are presented first (Table 1).

**Table 1:** Administrative information

1Title	** Effects of care of multimorbid patientes in general practices by advanced practice nurses: study protocol for a nonrandomized controlled trial**	
2a and 2b Trial registration	2a: Registry: DRKS00026172 on DRKS (German Clinical Trials Register) 2b: Data Set:	
	**Data category**	**Information**
	Primary registry and trial identifying number	DRKS00026172 on DRKS
	Date of registration in primary registry	2021/09/06
	Secondary identifying numbers	n/a
	Source(s) of monetary or material support	German Innovation Fund of the Federal Joint Committee
	Primary sponsor	German Innovation Fund of the Federal Joint Committee
	Secondary sponsor(s)	n/a
	Contact for public queries	Catholic University of Applied Sciences Mainz Prof. Dr. Renate Stemmer Saarstr. 3 55122 Mainz Germany Telephone: + 496131 28944 520 stemmer@kh-mz.de
	Contact for scientific queries	Catholic University of Applied Sciences Mainz Prof. Dr. Renate Stemmer Saarstr. 3 55122 Mainz Germany Telephone: + 496131 28944 520 stemmer@kh-mz.de
	Public title	Case-based care of multimorbid patients in general practice by advanced practice nurses (APNs)
	Scientific title	Effects of care of multimorbid patients in general practices by advanced practice nurses (FAMOUS): study protocol for a nonrandomized controlled trial
	Country of recruitment	Germany
	Health condition(s) or problem(s) studied	Multimorbidity
	Intervention(s)	Intervention group: GP-oriented care of multimorbid patients by APNs. Qualification profile of APNs: professional nursing licence, at least 2 years of professional experience in nursing, academic degree at master’s level, 500 hours project-specific advanced training. Task profile: in-depth assessment, preparation, monitoring and evaluation of a person-centred and evidence-based care plan. Control group: multimorbid patients receiving standard care.
	Key inclusion and exclusion criteria	Gender: all genders Minimum age: ≥ 18 years Maximum age: no maximum age Further inclusion criteria: medical diagnosis of at least three chronic diseases, which in the past 12 months in at least three out of four quarters have given rise to general practitioners’ treatment;being in general practitioners’ treatment; insurance with a statutory health insurance company. Exclusion criteria: not fulfilling the inclusion criteria; being in the dying phase according to the assessment of the general practitioner.
	Study type	Interventional, nonrandomized, nonblinded controlled trial
	Date of first enrolment	2021/10/01
	Target sample size	*n*=2451
	Recruitment status	Recruiting
	Primary outcome(s)	Number of emergency contacts
	Key secondary outcomes	Use of other health care facilities (e.g. hospital, nursing home), hospital readmission and visits by general practitioners
3 Protocol version	Issue Date: 2021/10/01	
4 Funding	There was financial support only, provided by the German Innovation Fund of the Federal Joint Committee. The Innovation Fund uses funds from the statutory health insurance (SHI) to support innovative, cross-sectoral new forms of care and patient-oriented care research projects in order to further develop and improve SHI-financed care in Germany	
5a Author details	RS and EG initiated the study design; CDS is responsible for the project management; JK developed the concept of the internal evaluation and will conduct it. EG and MB developed the concept of the external evaluation; BB and MB together with a statistician are planning and will conduct the statistical analysis. BB is responsible for the implementation of the qualitative interviews. All authors contributed to the development and refinement of the study protocol and approved the final manuscript.	
5b Name and contact information for the trial sponsor	Trial Sponsor: Catholic University of Applied Sciences Mainz (CUAS) Contact name: Prof. Dr. Renate Stemmer Address: 55122 Mainz, Saarstr. 3, Germany Telephone: + 49613128944520 Email: stemmer@kh-mz.de	
5c Role of funder	This funding source had no role in the design of this study and will not have any role during its execution, analyses, interpretation of the data, or decision to submit results.	

## Introduction

### Background and rationale

Multimorbidity is a common phenomenon among patients in general practices [1, 2]. The term multimorbidity is not well defined. However, most definitions characterize multimorbidity as the cooccurrence of two to three chronic diseases. Because of the somewhat unclear definition, the prevalence rate of multimorbidity in primary care patients varies across studies [2]. One systematic review [2] showed a prevalence of multimorbidity ranging from 12.9% in participants aged 18 years and older to 95.1% in a population aged 65 years and older. Worldwide, more than half of people over the age of 65 years suffer from multiple chronic conditions, most of which are treated in primary care [3]. Multimorbid patients experience many symptoms at the same time. Often, these symptoms cannot be attributed to a causal disease. Therefore, some key problems arise, including functional difficulties, polypharmacy, treatment burden, mental health difficulties, fragmentation of care, reduced quality of life and increased health care utilization [1]. Addressing these challenges requires optimizing functional capacity, rigorous medication management, a greater focus on patient-centred care, and collaboration across health care services [1]. The management of polypharmacy is complex [4], and the implementation of patient-centred care faces several barriers [5]. Thus, these goals can hardly be achieved in the short consultation time a general practitioner (GP) has, which is less than 10 minutes in Germany [6]. In addition, there is an increasing shortage of GPs, especially in rural areas. In Germany, there were already 3,300 unfilled primary care physician positions in 2019, and there will be approximately 11,000 in 2035 [7]. To address this issue, the involvement of other health care professionals is recommended [1]. In many countries, advanced practice nurses (APNs) are successfully integrated into primary health care for multimorbid patients [8, 9]. A systematic review of patient safety, clinical outcomes, cost, and patient satisfaction shows that care offered by nurse practitioners in primary health care settings provides equally good or better outcomes compared with GPs [10]. Outcomes regarding the use of resources, such as the number of emergency visits, hospital admissions, and outpatient contacts, are varied [11]. In addition, findings on the cost effectiveness of integrating APNs in primary care are not very clear. Some studies indicate no increase in costs [11], while other sources have found evidence for cost savings [12].

In Germany, outpatient medical care is predominantly provided by general practitioners. Unlike in many other countries, APNs are not deployed in general practices in Germany. One reason for this is that for a long time, there were no APN education opportunities at the master’s level in Germany. However, academically qualified nurses are now available thanks to the degree programs that have been established in Germany for some time. The objective of this study is to evaluate the care for multimorbid patients by APNs deployed in general practices in rural areas in Germany.

### Explanation for choice of comparators

To evaluate whether the integration of APNs in the primary care setting improves the care of multimorbid patients, a control group from German health care insurance was selected. Persons in the control group are considered to be receiving the current standard care. Therefore, a comparison between the new intervention and standard care is possible. To minimize the risk of confounding variables, participants in the intervention and control groups will be matched for the following variables: (1) age, (2) gender, (3) comorbidity and (4) living in a nursing home.

### Objectives

Our objective is to study whether the integration of APNs in the primary care of multimorbid patients leads to optimized care of the target group and to a reduction in the workload of the GPs. Improved care is expected to be reflected by a stabilization of the patients' health and home care situation. This will be expressed as the reduced use of emergency contacts (emergency services, emergency room, acute inpatient admission) and GP visits, thus reducing overall treatment costs. Finally, we expect to observe high satisfaction from all parties involved (physicians, patients, relatives, ANPs and medical assistants) with this type of care.

### Trial design

The FAMOUS trial is a prospective multicentre mixed methods study. Patients with at least three chronic conditions across nine participating general practices are eligible to be treated as an intervention group by a trained APN. All patients will be treated by APNs for twelve months. Data on the period of intervention are available as data from health insurance companies or the Association of Statutory Health Insurance Physicians (ASHIP) or data collected by the APN. The intervention group will be compared with a control group of patients from German health insurance who are matched according to age, sex, comorbidity and living in a nursing home. The matching will be performed at a ratio of one FAMOUS patient to two control patients.

Qualitative interviews will take place with GPs, APNs and patients to assess factors that are difficult to capture using quantitative data. This evaluation procedure will be carried out by an independent institution (IMBEI). In addition, the intervention will be assessed in more detail in an internal evaluation from the trial sponsor using a longitudinal design.

## Methods: participants, interventions and outcomes

### Study setting

The study is being conducted in nine general practices in rural areas of Rhineland-Palatinate, Germany. GPs interested in participating in the study were invited to apply and were selected by the project team of Catholic University of Applied Sciences (CUAS). The main criteria for selection were the number of suitable multimorbid patients and the willingness of the practice to implement the intervention. The number of treatment cases in the participating general practices ranges between 2000 and 6000 patients per quarter. Approximately 30% of them are multimorbid. With the size of the practice, we have ensured that the number of potentially eligible patients is sufficient to achieve the number of cases targeted in the study.

### Eligibility criteria

Patients (or a legal representative) must provide written informed consent before any study procedures occur. The inclusion criteria are as follows: at least 18 years of age; medical diagnosis of at least three chronic diseases that have led to GP treatment in at least three out of four quarters in the last twelve months; and insured by statutory health insurance. The exclusion criterion is a life expectancy less than 12 months as assessed by the treating physician.

### Interventions

#### Intervention description

The intervention aims to integrate person-centred and evidence-based APN care for multimorbid patients in general practices in rural areas in Germany. To be eligible for participation, the general practice must confirm its willingness to change some structural and process routines to integrate APN work into its workflow. Examples include providing a treatment room and a car for home visits as well as ensuring weekly case conferences to support APN work.

The qualification profile of APNs includes a professional nursing licence, at least two years of professional experience in nursing and an academic degree at the master’s level in a nursing-related program. In addition, APNs complete 500 h of project-specific training.

Within the scope of the study, APNs take over the care of multimorbid patients. The integration of APN care for multimorbid patients in practice is a complex intervention [13]. The complexity arises for various reasons. First, multimorbidity is defined by the number of diseases without further delineation, which means that the diseases and their combinations can vary greatly from one individual to another. The varying care needs lead to individual care goals. To achieve these different goals, APNs must take on a number of possible roles depending on the needs of the patients, and their activities must be selected and implemented flexibly.

To stabilize the individual health care situation, APNs implement the care process, establish or improve a supportive network and intensify the communication between all those involved in the provision of care. Furthermore, APNs actively involve patients in the care process and specifically promote patient autonomy. Conducting the care process means developing, carrying out, monitoring and evaluating a person-centred and evidence-based care plan.

APNs generally begin the care process with an in-depth assessment of cognitive capabilities, nutritional status, pain status, mobility status and psychosocial status. In the first step, cognitive performance is checked with a Dementia Detection test (DemTect) [14]. Depending on the result, different instruments or different data sources are used. If the DemTect score is above eight points, the following instruments are used: The Mini Nutritional Assessment (MNA) [15], Numeric Rating Scale (NRS) [16], Pain Disability Index (PDI) [17], Health Assessment Questionnaire (HAQ) [18], and Mannheim Inventory of Living Conditions in Old Age [Mannheimer Inventar der Lebensverhältnisse im Alter (MILVA)] [19]. In such cases, the data source is represented by the self-statements of the patients. If the DemTect score is less than or equal to eight points, the MNA and MILVA are still used but are assessed based on third-party observation, mainly by relatives. To measure the presence or extent of pain in this group, we use the Pain Assessment in Advanced Dementia Scale (PAINAD-G) [20, 21]. The De Morton Mobility Index (DEMMI) [22] is used to assess mobility. In addition, a clinical assessment is performed. Based on the values of the assessment instruments and the clinical assessment, a needs assessment is prepared, which forms the basis for the care plan. Taking the patient's individual needs and requirements into account, APNs plan specific interventions together with the patients and their relatives, as appropriate, to improve the patient's overall health and autonomy. In doing so, they implement the principle of shared decision-making. The interventions of the care plan are aimed at stabilizing or improving physical health, such as mobility, nutrition, neuropsychiatric state, continence status, skin condition/wounds, pain status and medication management, psychosocial situation, self-management skills, home living situation and social situation around loneliness. Where possible, the APNs involve family members and other related parties in the provision of care. In addition, the APNs include other stakeholders from the health care sector, such as outpatient care services, care advice centres, physiotherapists, and occupational therapists, or initiate further support services, such as volunteer visitation services or citizens’ buses.

The initial assessment takes place in the patient's home. Depending on the patient's condition, this initial examination is performed over one or two visits. Due to the patient’s condition and to his or her often reduced mobility, most of the follow-up visits also take place in the patient’s home. If possible, patients are seen at the APN consultation hours, which is held in the rooms of the GP ‘s office. All patients receive a business card with the APN's contact information so they can contact them by phone or email between scheduled visits if needed.

Weekly case conferences are scheduled for consultations with the GP. If necessary, short-term consultations are possible at any time. In the regular case conferences, APNs and GPs discuss the health status and the care situation of the participating patients. APNs make suggestions for further treatments, while the GPs decide on the medical procedure. Following German law, the medical side of care is provided under the delegation model.

#### Modifications

The range of possible activities of APNs is broad and flexible. A deviation from the protocol could arise if a patient wishes to be cared for by the physician instead of the APN more than actually intended, thus preventing APNs from fulfilling their potential. In this case, the primary care physician will be involved in the care as needed.

#### Adherence

The introduction of APN roles and APN care in primary care practices is new for everyone involved. Including APNs, primary care physicians and patients. The primary burden for this implementation process falls on APNs, but physicians are also involved. Therefore, pioneer APNs are supervised by experienced mentors during their role development process in primary care. This mentoring consists of weekly short phone calls and monthly structured evaluation interviews. In addition, mentors conduct quarterly structured interviews with primary care physicians. Depending on the needs that become clear in the various conversations, supportive activities by the mentors can follow, with the aim of ensuring the implementation of the planned intervention.

#### Concomitant care

Based on patient needs, other health care providers besides the APN and GPs (e.g., physiotherapists, medical specialists) might be needed, which is accepted in this study.

### Outcomes

#### Primary endpoint

The primary endpoint is the number of emergency contacts per patient within one year. Emergency contacts are visits to the on-call medical service, GP contacts outside office hours and unplanned hospital admissions.

#### Secondary endpoints

The secondary endpoints in question are the use of continuing care institutions (inpatient hospitalizations, specialist visits, and nursing home stays), hospital readmission and home visits by primary care physicians. In addition, the expectations and experiences of participating physicians, APNs, and patients are mapped, and the financial impact of the intervention will be measured. The primary and secondary endpoints will be assessed by the IMBEI.

Outcomes of the internal evaluation are the health status of the patients and the satisfaction of physicians, patients and relatives with the APN care. In addition, the role development of the APNs and the experiences of the medical assistants are evaluated.

### Participant timeline

The initial data of the participating patients includes age, sex, medical diagnoses, and number and duration of illnesses. These data are collected during the enrolment phase. Each patient enrolled in the project takes part in APN care for twelve months. The initial assessment lasts on average 1.5 hours. Follow-up contacts for the implementation and continuation of the care plan usually take place every four weeks. Depending on the need, however, the intervals can also be significantly shorter or significantly longer. APN contact is held at least quarterly. Routine contacts last on average 30 minutes. Depending on patient concerns and needs, the time frame may vary.

Routine data are used to count emergency contacts as the primary outcome measure, which means that there is no time stress for the patients. Secondary data are supplied by the ASHIP and by one health insurance company. However, there are patients for whom not all data regarding hospital and emergency contacts are available from routine data. For them, data are collected by the APNs. These data are also used for the analysis of secondary endpoints and health economic analyses. In addition, 30 patients will be interviewed about their experiences after completion of the 12-month intervention. We do not foresee the need for follow-up data collection.

The assessment tools mentioned above are used to measure the health status of the patients in the internal assessment. Data will be collected at the beginning (t0) and at the end (t1) of the 12-month intervention. Patient satisfaction with APN care is measured using a project-specific questionnaire.

The participants’ timeline in relation to the individual steps that occur during enrolment, intervention and evaluation is shown in Table 2:

**Table 2:**
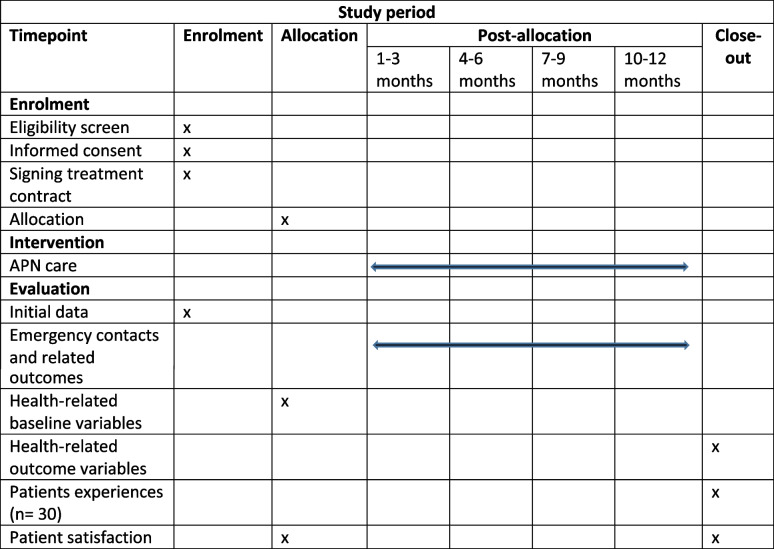
Participant timeline

### Sample size

The number of cases was calculated on the basis of the number of emergency contacts after twelve months (primary outcome). Based on literature estimates, it was assumed that the expected number of 2.7 emergency contacts per patient per year could be reduced by approximately 15% to 2.295 emergency contacts. The alpha was assumed to be 0.05. Taking into account the cluster effect and a dropout of 30% and a target power of 80%, this results in 817 patients in the intervention group and 1634 patients in the control group.

### Recruitment

Recruitment of patients will be carried out by the participating general practitioners through a direct approach. Patients trust their GPs, so a recommendation to participate made by primary care physicians is likely to result in a high participation rate. The recruitment process is supported by the offer of personal contact between APNs and potential participants in the enrolment phase. In addition, to support recruitment, the patients are provided with comprehensive information and educational material about the project in easy-to-understand language, informing them about the project, its objectives and the data collection and processing that will be carried out to achieve the project goals. Posters and flyers are placed in the waiting rooms of the participating GP offices and are customized with the individual data of the general practice and the APN.

## Methods: assignment of interventions

The study reported here is a nonrandomized and nonblinded study. This means that the issues on allocation and blinding are not applicable.

## Methods: data collection, management, analysis

### Data collection methods

For the independent evaluation, data from ASHIP and the health insurance fund will be used for primary and secondary endpoints. To fill in missing data, such as hospital visits for patients with other health insurance, APNs collect health-related data and document patients' emergency and routine visits to any health-related facility (doctor's office, hospital, emergency room). The health insurer provides a data pool for control group matching. All data are transferred to the IMBEI in pseudonymized form. Patients, GPs, and APNs are informed by mailed invitations about the possibility of interview participation and asked to complete a feedback form. Potential interviewees are called via the feedback form, and appointments are made. The qualitative interviews are conducted by telephone using a previously prepared standardized interview guide. There is a separate guide for each interview group, which is finalized after piloting. Interviews are audio recorded and transcribed verbatim.

As part of the internal evaluation, the health status of each participating patient was measured before (t0) and at the end of the patient-individual intervention phase (t1) using the assessment instruments mentioned in the intervention section above as we use the data from these instruments for care and research purposes. Their psychometric data are as follows: DemTect: interrater reliability *r* = 0.993, sensitivity 85.1%, specificity 97% [14]; MNA: internal consistency α = 0.701, test–retest reliability *r* = 0.697 [23]; NRS: with a cut-off point NRS > 4, the sensitivity of ‘unbearable pain’ is 83%, while the specificity is 96.7%, with a cut-off point NRS > 5, the sensitivity is 94%, while the specificity is 85% [24]; PDI: internal consistency α = 0.86, [17]; HAQ: internal consistency α = 0.78–0.98 [25, 26]. The psychometric results of the German translation are as follows: test–retest reliability 0.94, internal consistency α = 0.92, criterion validity 0.76 [27]; MILVA: internal consistency range between α = 0.61–0.94, sensitivity 72%, specificity 59%; [17]. PAINAD-G: internal consistency α = 0.85, interrater stability *r* = 0.80, retest reliability* r* = 0.90 [21]; DEMMI: internal consistency α = 0.83, interrater reliability ICC 0.94 [28].

For the internal evaluation of patients’ satisfaction with APN care, a standardized questionnaire is used before and at the end of the patient-individual intervention phase. We take the APN-BQ (Advanced Practice Nurse- counselling quality) questionnaire and modify it for project-specific concerns. The APN-BQ aims to measure the quality of advanced practice nurse counselling in home care settings. The APN-BQ has shown itself to be a reliable instrument with good content and construct validity. The value is α = 0.86 [29]. The questionnaire for relatives was adapted to the patient questionnaire, which made sense from a thematic point of view. The timing of the data collection runs parallel to the data collection from the patients. All GPs in the participating practices are questioned via standardized project-specific questionnaires at the beginning (t0) and the end (t1) of the project intervention. In addition, qualitative interviews are conducted with medical assistants as well as with the APNs. Two medical assistants from each participating general practice are interviewed before (t0) and at the end of the 24-month period of project intervention (t1), and all nine APNs are interviewed before (t0), after 12 (t1) and after 24 months (t2) of intervention. The interviews of both groups are guideline based.

### Data management

Data from patients in the intervention group will be entered by the APNs. For this purpose, a special secure software that stores all data securely and provides secure data transfer to the trust centre has been developed. The IMBEI receives the pseudonymised data from the trust centre as well as from the health insurance funds and the ASHIP by means of Qiata File Transfer Appliances. All data, which have already been checked and cleaned by the ASHIP and the health insurance fund, as well as the data of the APN entries, are again subjected to a quality and plausibility check by the IMBEI.

Both quantitative and qualitative data received by the IMBEI are stored in a secure network at the University Medical Center of the Johannes Gutenberg University Mainz. The appropriate technical and organizational measures according to the General Data Protection Regulation (GDPR) are listed in a separate document. Upon completion of the project, all data will be archived and secured in accordance with applicable laws.

### Statistical and further methods

#### Statistical and further methods for primary and secondary outcomes

Statistical analyses of the independent evaluation take place as a comparison between intervention and control groups. Poisson regression will be used for both the primary and secondary endpoints. Adjustments are made for relevant confounders but not for matching variables. APN practices in the intervention group are not considered clusters because a comparable cluster variable is not available for the control group.

Users of the new form of care (patients, participating primary care physicians, and APNs) will be interviewed in qualitative interviews. The interviews will take place at the end of the intervention period to reflect the experiences made. Additionally, primary care physicians will be interviewed at the beginning of the project to map expectations of the new form of care. It can be assumed that a theoretical saturation – i.e., the point at which new interviews no longer provide additional information – will be reached with *n* = 5 GPs and *n* = 5 APNs. Full data collection from all 9 practices participating in the project will nevertheless be targeted. For patients, a number of *n* = 30 participants is expected to reach theoretical saturation.

Cost analysis from the social health insurance perspective will be performed to evaluate potential savings. Costs will be compared between the intervention and the control group. Total cost analysis and cost analysis within subgroups will be carried out to identify areas with the highest costs or cost savings. These subgroups will be identified through the primary and secondary endpoints. Data for the health economic analysis will be obtained from the participating health insurance. Intangible or nonmedical costs will not be analysed since these data are not available in the insurance dataset.

In the internal evaluation, descriptive and analytical statistical methods are used. The patient’s situation at the beginning and at the end of the APN intervention and the nursing process will be described by statistical indicators such as percentual distribution, mean and standard deviation or median and quartile deviation. Depending on the kind of data and on its distribution, parametric or nonparametric statistical analyses will be conducted (e.g., variance analysis, t test, Mann-Whitney U test or Wilcoxon test).

### Additional analyses

Additional analyses will take place for the individual endpoints, which are combined into the aggregate endpoint of emergence contacts. Subgroup analyses as well as sensitivity analyses will be performed.

### Analysis population and missing data

The data for the analyses come from different sources. The data of the control group and the data of the insured persons of the participating health insurers are assumed to be complete. Data from the ASHIP are available for all patients. Therefore, it can be assumed that the outpatient care data of all patients are complete. Inpatient data of patients who are not insured by the participating health insurance company will be requested from the APN and entered into a database. Subgroup analyses were performed to assess the data quality of these data. Data can be supplemented by means of imputation procedures.

## Monitoring methods

### Data monitoring

Due to the low risk, no data monitoring committee (DMC) will be established. Interim analyses will not be conducted.

### Harms

Adverse events are not to be expected. APNs will be in regular contact with patients, so they should be able to detect any deterioration in their health status. If the APNs notice any unclear change in the health status of the patients, they will discuss this with the GPs. In addition, the GPs have regular contact with the patients. If any adverse event should occur, the APN will report to the mentor accompanying her or him.

### Auditing

The routine data provided by the health insurance company and ASHIP are controlled and quality-checked through well-established procedures. These high control standards are necessary beyond the research requirements because routine data are usually the basis for billing modalities.

The documentation form, which is used to document the patients' health status and the activities of the APNs, is continuously monitored by the experienced mentors who accompany the APNs. In the monthly structured evaluation meetings or - if necessary - in between, the mentors provide the APNs with feedback and discuss any need for improvement.

Mentors are part of the project team, which holds weekly meetings to discuss the progress of the project and to make steering decisions as necessary.

## Ethics and dissemination

### Research ethics approval

The protocol, informed consent forms, participant education and recruitment materials, and other requested documents were approved by the Ethics Committee of the German Society of Nursing Science. The ethical reference issued (No. 21–012). All methods were carried out in accordance with relevant guidelines and regulations. Informed consent was obtained from all subjects or their legal guardian.

### Protocol amendments

Any change to the protocol that may affect the conduct of the study, potential benefit to patients, or patient safety, including changes in study objectives, study design, patient population, sample size, study procedures, or significant administrative aspects, will require a formal amendment to the protocol. Such a change will be submitted to the Ethics Committee of the German Society for Nursing Science and will only be implemented if approved.

Administrative changes to the protocol are minor corrections and/or clarifications that do not affect the conduct of the study. The Ethics Committee of the German Society for Nursing Science will be informed about these administrative changes.

### Consent or assent

Patients who meet the inclusion criteria are informed verbally and in writing by their GP. If desired, patients will have the opportunity to speak with their potential future APN. She or he will explain the APN care program again. General practitioners obtained informed consent from patients if they decided to participate in the study. Participants document their informed consent by signing a treatment contract. If necessary, the information is passed on to the legal representatives, and their signature on the treatment contract is obtained.

### Confidentiality

In accordance with the GDPR and the project-specific data privacy concept, all study-related information is kept securely at the study site. All participant information is kept in locked file cabinets. All reports, data collection, process, and administrative forms will be marked with a coded ID (identification number) only to maintain participant confidentiality. All records containing names or other personal identifiers, such as consent forms, are kept separate from study records, which are marked with a code number. All local databases are secured by a password protected access system. The health record consists of two parts: part one contains the data identifying the person, and part two contains the health-related data. The person-identifying data and the health-related data are hosted on different servers. Therefore, only pseudonymized data are made available to the external evaluator.

### Declaration of interests

All authors have completed the International Committee of Medical Journal Editors (ICMJE) uniform disclosure form at http://www.icmje.org/disclosure-of-interest/ and declare that they have no support from any organization for the submitted work (with the exception of the funding by the Innovation Fund); no financial relationships with any organizations that might have an interest in the submitted work in the previous three years; and no other relationships or activities that could appear to have influenced the submitted work.

### Access to data

The Principal Investigator will have access to the cleaned datasets by the independent evaluator. All other partners involved in the project may have access to the data upon request. There are no contractual agreements restricting this access.

### Ancillary and posttrial care

During the intervention phase, care is provided by the APN within the framework of delegation. This means that the physicians are informed at all times about the patient's state of health and the treatment steps. After the end of the intervention, the patients returned to the normal care of general practice.

### Dissemination policy

The study group aims to publish the results in scientific papers, present them at scientific congresses and use all appropriate channels from a scientific point of view to inform the scientific community. In addition, the study group will inform policy-makers and health care providers involved in this area about the outcomes. The various partners of the study group will be informed in advance of planned publications as contractually agreed upon.

The main publication will be prepared by the study director. All active project partners are coauthors, according to the guidelines of the ICMJE [30].

All partners involved in the project are allowed to publish the data obtained. To do so, they must first prepare an exposé that is submitted to the Publication Steering Committee, which is composed of the study director and the head of the independent evaluation. If the exposé is approved, an application for data export can then be submitted to the IMBEI. All publications must be submitted to and approved by the FAMOUS Publication Steering Committee prior to submission.

## Data Availability

Under the assumption that national data protection requirements are fully met, access to aggregated or pseudonymized individual-level data may be made available upon reasonable request. All data access requests should be directed to the corresponding author.

## Abbreviations

APN: Advanced Practice Nurse
APN-BQ: Advanced Practice Nurse- counselling quality [Beratungsqualität]
ASHIP: Association of Statutory Health Insurance Physicians
CUAS: Catholic University of Applied Sciences
DEMMI: De Morton Mobility Index
DemTect: Dementia Detection
DMC: Data Monitoring Committee
DRKS: German Clinical Trials Register [Deutsches Register Klinischer Studien]
FAMOUS: Case-based care of multimorbid patients in general practice by advanced practice nurses [Fallbezogene Versorgung multimorbider Patient/innen in der Hausarztpraxis durch Advanced Practice Nurses]
GDPR: General Data Protection Regulation
GP: General Practitioner
HAQ: Health Assessment Questionnaire
ICC: Intra-Class Correlation coefficient
ICMJE: International Committee of Medical Journal Editors
IMBEI: Institute of Medical Biostatistics, Epidemiology and Informatics
IZKS: Interdisciplinary Center Clinical Trials
MILVA: Mannheim Inventory of Living Conditions in Old Age [Mannheimer Inventar der Lebensverhältnisse im Alter]
MNA: Mini Nutritional Assessment
n/a: Not applicable
NRS: Numeric Rating Scale
PAINAD-G: Pain Assessment in Advanced Dementia Scale
PDI: Pain Disability Index
SHI: Statutory Health Insurance
